# Association between female reproductive factors and gout: a nationwide population-based cohort study of 1 million postmenopausal women

**DOI:** 10.1186/s13075-021-02701-w

**Published:** 2021-12-16

**Authors:** Yeonghee Eun, In-Young Kim, Kyungdo Han, Kyu Na Lee, Dong-Yun Lee, Dong Wook Shin, Seonyoung Kang, Seulkee Lee, Hoon-Suk Cha, Eun-Mi Koh, Jaejoon Lee, Hyungjin Kim

**Affiliations:** 1 Department of Medicine, Samsung Medical Center, Sungkyunkwan University School of Medicine, 81 Irwon-Ro, Gangnam-gu, Seoul, 06351 South Korea; 2 Department of Medicine, National Police Hospital, Seoul, South Korea; 3 Department of Statistics and Actuarial Science, Soongsil University, Seoul, South Korea; 4 Department of Biomedicine and Health Science, The Catholic University of Korea, Seoul, South Korea; 5 Department of Obstetrics and Gynecology, Samsung Medical Center, Sungkyunkwan University School of Medicine, Seoul, South Korea; 6 Department of Family Medicine and Supportive Care Centre, Samsung Medical Center, Sungkyunkwan University School of Medicine, Seoul, South Korea; 7 Department of Clinical Research Design and Evaluation/ Department of Digital Health, Samsung Advanced Institute for Health Science and Technology (SAIHST), Sungkyunkwan University, Seoul, South Korea; 8 Department of Medical Humanities, Samsung Medical Center, Sungkyunkwan University School of Medicine, 81 Irwon-Ro, Gangnam-gu, Seoul, 06351 South Korea

**Keywords:** Crystal arthropathies, Reproductive, Epidemiology, Hormones, Pregnancy and rheumatic disease

## Abstract

**Background:**

Previous studies have shown that the incidence and risk factors of gout differs according to sex. However, little research has been done on the association between reproductive factors and gout. We conducted an analysis of a large nationwide population-based cohort of postmenopausal women to determine whether there is an association between reproductive factors and the incidence of gout.

**Methods:**

A total of 1,076,378 postmenopausal women aged 40–69 years who participated in national health screenings in 2009 were included in the study. The outcome was the occurrence of incident gout, which was defined using the ICD-10 code of gout (M10) in the claim database. Cox proportional hazard models were used for the analyses and stratified analyses according to body mass index (BMI) and the presence/absence of chronic kidney disease (CKD) were performed.

**Results:**

The mean follow-up duration was 8.1 years, and incident cases of gout were 64,052 (incidence rate 7.31 per 1000 person-years). Later menarche, earlier menopause, and a shorter reproductive span were associated with a high risk of gout. No association between parity and gout incidence was observed. Use of oral contraceptives (OC) and hormone replacement therapy (HRT) were associated with an increased risk of gout. The association between reproductive factors and gout was not statistical significant in the high BMI group. The effects of OC and HRT usage on gout were not significant in the CKD group.

**Conclusion:**

Shorter exposure to endogenous estrogen was associated with a high risk of gout. Conversely, exposure to exogenous estrogen such as OC and HRT was associated with an increased risk of gout.

**Supplementary Information:**

The online version contains supplementary material available at 10.1186/s13075-021-02701-w.

## Introduction

Gout is the most common inflammatory arthritis in adults, and its incidence has constantly increased over recent decades [1]. Gout is 3–10 times more common in men than in women [2]. However, the incidence of gout increases in postmenopausal women, whereas premenopausal women are protected by the uricosuric effect of estrogen [3, 4].

Previous epidemiologic studies have investigated the association between reproductive factors and gout. A cross-sectional analysis of 1530 women had shown that postmenopausal status, earlier age at menarche, and history of oral contraceptive (OC) use were associated with high serum uric acid concentration [5]. A retrospective case-control study of 13,489 female incident gout patients older than 45 years of age had shown that current use of opposed estrogen was associated with a low odds ratio of incident gout [6]. In the Nurses’ Health Study, early menopause was associated with an increased risk of incident gout, and hormone replacement therapy (HRT) was associated with a decreased risk of gout [7]. However, there have been conflicting results reported in other studies. In a study published in 2008 that had analyzed data from the US National Health and Nutrition Examination Survey (NHANES) from 1988 to 1994, it had been reported that menopause was associated with higher serum uric acid levels, while HRT was associated with lower uric acid levels [8]. In contrast, a study that had analyzed NHANES data from 1999 to 2010 had stated that the prevalence of hyperuricemia was not related to menopause or current use of HRT [9].

As shown, the results on the association between gout and female reproductive factors are largely inconclusive. Among the studies to date, the largest sample size had been 120,000 subjects [7], and many studies had been conducted in a case-control or cross-sectional manner [6–9]. In addition, various reproductive factors had not been included. In pre- and postmenopausal women, the effects of reproductive factors on disease occurrence may differ [10], and variables such as age at menopause, reproductive span, and HRT may only be evaluated in postmenopausal women. Therefore, in this study, we **i**nvestigated the association between various female reproductive factors and the incidence of gout in a nationwide population-based cohort of postmenopausal women.

## Methods

### Data resource

The Korean National Health Insurance Service (NHIS) is a government insurer covering nearly 97% of the population living in Korea. The remaining 3% of the population, who are in the lowest income bracket, are also covered by NHIS through a medical aid program. The NHIS database includes information on sociodemographic variables, health care utilization, health screening, and deaths among the entire population of South Korea [11, 12]. The NHIS provides health screening to all Koreans older than 40 years of age and all employees regardless of age every other year, through which health screening information is collected. Also, as part of the National Cancer Screening Program (NCSP), the NHIS provides breast cancer screening every other year to all women older than 40 years of age. Women participating in the breast cancer screening program must respond to a questionnaire regarding their reproductive history, which is collected by the NHIS. The NHIS database and its health screening information had been widely used in earlier epidemiologic studies to study the effects of female reproductive factors on various diseases [13, 14].

This study complies with the Declaration of Helsinki and was approved by the Institutional Review Board of Samsung Medical Center (IRB File No. SMC 2021–01-011), who waived the requirement for written informed consent of the study subjects, because their data were publicly available and anonymized.

### Study population

**Fig. 1:**
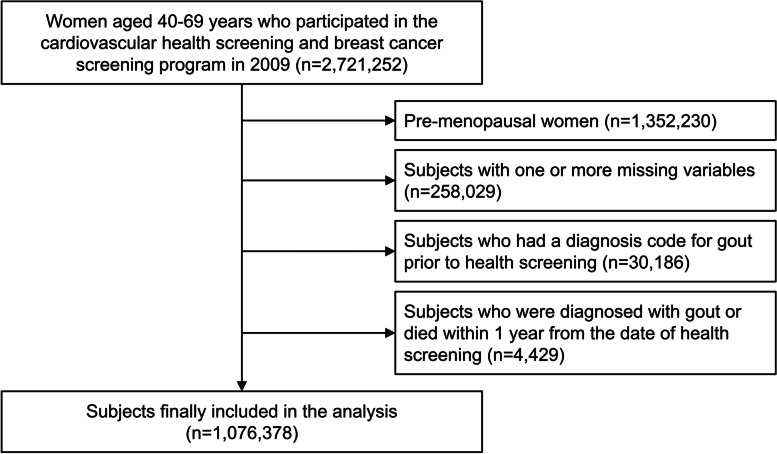
Flowchart of the study population

Of the 2,721,252 women aged 40–69 years who participated in the cardiovascular health and breast cancer screening program in 2009, 1,369,022 were postmenopausal. Among them, subjects with one or more missing data for the variables of interest (*n* = 258,029), subjects who had been diagnosed with gout (International Classification of Disease 10th revision [ICD-10] code M10) before medical examination (*n* = 30,186), and subjects who had been diagnosed with gout or who had died within 1 year of the health screening date (*n* = 4429) were excluded from the study. A total of 1,076,378 subjects were finally included in the analysis (Fig. 1).

### Study outcomes and follow-up

The endpoint of the study was incident gout, defined as two outpatient visits or one hospitalization with the diagnostic code of gout (ICD-10 code M10). The cohort was followed-up from 1 year after the health screening date until the date of outcome occurrence, death, or the end of the study (December 31, 2018), whichever came first.

### Data collection

Information on health-related behaviors and reproductive factors was collected through a self-administered questionnaire during health screening and breast cancer screening programs. Age at menarche and age at menopause were collected as continuous variables. Age at menarche was categorized as ≤12 years, 13–14 years, 15–16 years, or > 16 years, and age at menopause was categorized as < 40 years, 40–44 years, 45–49 years, 50–54 years, or ≥ 55 years. Each variable was categorized according to the distribution of the Korean women. Reproductive span was calculated as the interval between age at menarche and age at menopause. The duration of OC use was categorized as never, < 1 year, or ≥ 1 year, and the duration of HRT use was categorized as never, < 2 years, 2–5 years, or ≥ 5 years. Parity, breastfeeding, OC, and HRT were categorized according to the classification in the questionnaire. Parity was categorized as 0 children, 1 child, or ≥ 2 children. The total duration of breastfeeding was categorized as never, < 6 months, 6–12 months, or ≥ 12 months.

Smoking status was classified as never, ex-, or current smoker. Alcohol consumption was classified as none (0 g/day), mild (< 30 g/day), or heavy (≥30 g/day). Regular exercise was defined as moderate physical activity for ≥30 min at least 5 times per week, or vigorous physical activity ≥20 min at least 3 times per week. Body mass index (BMI) was calculated using the body weight and height measured at the health examination, and was categorized as underweight (< 18.5 kg/m^2^), normal (18.5–23 kg/m^2^), overweight (23–25 ​​kg/m^2^), obese (25–30 kg/m^2^), or severely obese (≥30 kg/m^2^) according to the Asia-Pacific criteria of the World Health Organization. Estimated glomerular filtration rate (eGFR) was calculated using the Modification of Diet in Renal Disease formula using the creatinine measured on the day of the health examination and the patient’s age at the time. Chronic kidney disease (CKD) was defined as an eGFR of < 60 mL/min/1.73 m^2^, and baseline comorbidities such as hypertension, diabetes, and hyperlipidemia were identified through a combination of the past medical history section of the questionnaire, ICD-10 code, and prescription history. Income level was categorized into quartiles based on information of income-based premiums charged by health insurance.

### Statistical analysis

Continuous variables with normal distribution were expressed as mean ± standard deviation (SD), and categorical variables were expressed as numbers and percentages. The incidence rate of gout was calculated by dividing the number of incident cases by the total follow-up period. Cox proportional hazard models were used to calculate hazard ratios (HRs) and 95% confidence intervals (CIs) for the risk of incident gout according to various reproductive factors. Model 1 was a non-adjusted model, and Model 2 was a multivariable model including age, age at menarche, age at menopause, duration of OC use, duration of HRT, parity, duration of breastfeeding, BMI, smoking, alcohol consumption, regular exercise, hypertension, diabetes mellitus, hyperlipidemia, chronic kidney disease, and income. For the analysis of the reproductive span, a modified version of Model 2 – in which age at menarche and age at menopause were replaced by reproductive span – was used. To examine the effect of each reproductive factor on gout, separate analyses according to the temporal order between the reproductive factors were performed, since the specific causal structure between the reproductive factors is not yet clearly known. Statistical significance was set at *p* < 0.05. All statistical analyses were performed using SAS version 9.4 (SAS Institute Inc., Cary, NC, USA).

## Results

### Baseline characteristics

The baseline characteristics of the study population stratified by the presence/absence of gout are presented in Table 1. The mean age of the study subjects who had gout and without gout was 59.0 years and 58.5 years, respectively. Women diagnosed with gout had a higher BMI and a higher percentage of comorbidities than women without gout. Although the differences were not significant (standardized mean difference < 0.1), subjects diagnosed with gout had late menarche, early menopause, and short reproductive span, and had higher rates of use of OC, HRT, and breastfeeding for ≥1 year compared to subjects without gout.

**Table 1:** Baseline characteristics of study population stratified by the presence/absence of gout

Variable	Women without gout (***n*** = 1,012,326)	Women with gout (***n*** = 64,052)	***p***-value	SMD
Age	58.5 ± 5.8	59.0 ± 5.8	<.0001	0.0829
Body mass index (kg/m^2^)	24.2 ± 3.1	24.8 ± 3.3	<.0001	**0.1813**
< 18.5	17,726 (1.8)	781 (1.2)	<.0001	0.0440
18.5–23	350,304 (34.6)	18,395 (28.7)		**0.1268**
23–25	272,058 (26.8)	16,682 (26.0)		0.0188
25–30	329,416 (32.5)	24,088 (37.6)		**0.1063**
≥ 30	42,822 (4.2)	4106 (6.4)		0.0973
Smoking			<.0001	
Never	975,659 (96.4)	61,100 (95.4)		0.0497
Ex-smoker	10,389 (1.0)	790 (1.2)		0.0196
Current smoker	26,278 (2.6)	2162 (3.4)		0.0458
Alcohol consumption			<.0001	
None	874,134 (86.4)	55,078 (86.0)		0.0104
Mild (< 30 g/day)	132,404 (13.1)	8497 (13.3)		0.0055
Heavy (≥30 g/day)	5788 (0.6)	477 (0.7)		0.0214
Regular exercise	201,742 (19.9)	12,626 (19.7)	0.1834	0.0054
Comorbidities				
Hypertension	410,364 (40.5)	31,386 (49.0)	<.0001	**0.1708**
Diabetes mellitus	114,463 (11.3)	10,568 (16.5)	<.0001	**0.1505**
Hyperlipidemia	338,200 (33.4)	25,238 (39.4)	<.0001	**0.1248**
Chronic kidney disease	89,624 (8.9)	8226 (12.8)	<.0001	**0.1286**
Creatinine (mg/dL)	0.87 ± 0.86	0.90 ± 0.90	<.0001	0.0356
Estimated GFR (mL/min/1.73 m^2^)	83.4 ± 26.8	81.3 ± 27.3	<.0001	0.0754
Income (quartile)			<.0001	
Q1 (lowest)	241,520 (23.9)	15,594 (24.4)		0.0114
Q2	196,934 (19.5)	12,646 (19.7)		0.0073
Q3	258,906 (25.6)	16,797 (26.2)		0.0148
Q4 (highest)	314,966 (31.1)	19,015 (29.7)		0.0310
Age at menarche (years)	16.3 ± 1.8	16.4 ± 1.8	<.0001	0.0376
≤ 12	11,212 (1.1)	635 (1.0)	<.0001	0.0114
13–14	137,691 (13.6)	8329 (13.0)		0.0176
15–16	405,514 (40.1)	24,984 (39.0)		0.0215
> 16	457,909 (45.2)	30,104 (47.0)		0.0354
Age at menopause (years)	50.2 ± 3.9	50.2 ± 4.0	<.0001	0.0171
< 40	14,663 (1.5)	1091 (1.7)	<.0001	0.0205
40–44	50,330 (5.0)	3412 (5.3)		0.0161
45–49	273,603 (27.0)	17,389 (27.2)		0.0027
50–54	562,497 (55.6)	34,968 (54.6)		0.0195
≥ 55	111,233 (11.0)	7192 (11.2)		0.0077
Reproductive span (years)	33.9 ± 4.2	33.7 ± 4.4	<.0001	0.0318
< 30	121,110 (12.0)	8259 (12.9)	<.0001	0.0282
30–34	409,702 (4057)	26,066 (40.7)		0.0046
35–39	413,718 (40.9)	25,375 (39.6)		0.0255
≥ 40	67,796 (6.7)	4352 (6.8)		0.0039
Oral contraceptives (years)			<.0001	
Never	842,604 (83.2)	52,498 (82.0)		0.0336
< 1	103,252 (10.2)	6846 (10.7)		0.0160
≥ 1	66,470 (6.6)	4708 (7.4)		0.0308
Hormone replacement therapy (years)			<.0001	
Never	826,017 (81.6)	51,071 (79.7)		0.0472
< 2	108,676 (10.7)	7508 (11.7)		0.0313
2–5	44,509 (4.4)	3075 (4.8)		0.0193
≥ 5	33,124 (3.3)	2398 (3.7)		0.0256
Parity			0.0575	
0 children	26,722 (2.6)	1700 (2.7)		0.0098
1 child	69,017 (6.8)	4210 (6.6)		0.0079
≥ 2 children	916,587 (90.5)	58,142 (90.8)		0.0009
Breastfeeding (months)			<.0001	
Never	74,885 (7.4)	4619 (7.2)		0.0383
< 6	74,108 (7.3)	4070 (6.4)		0.0273
6–12	187,857 (18.6)	11,213 (17.5)		0.0472
≥ 12	675,476 (66.7)	44,150 (68.9)		0.0072

Continuous data with normal distribution are expressed as the mean ± standard deviation, and categorical data are expressed as n (%)

*SMD* standardized mean difference, *GFR* glomerular filtration rate, *Q* quartile

### Association between incident gout and endogenous estrogen related factors

The mean follow-up duration was 8.14 ± 1.2 years. During the study period, 64,052 people were newly diagnosed with gout (incidence rate 7.31/1000 person-years).

**Fig. 2:**
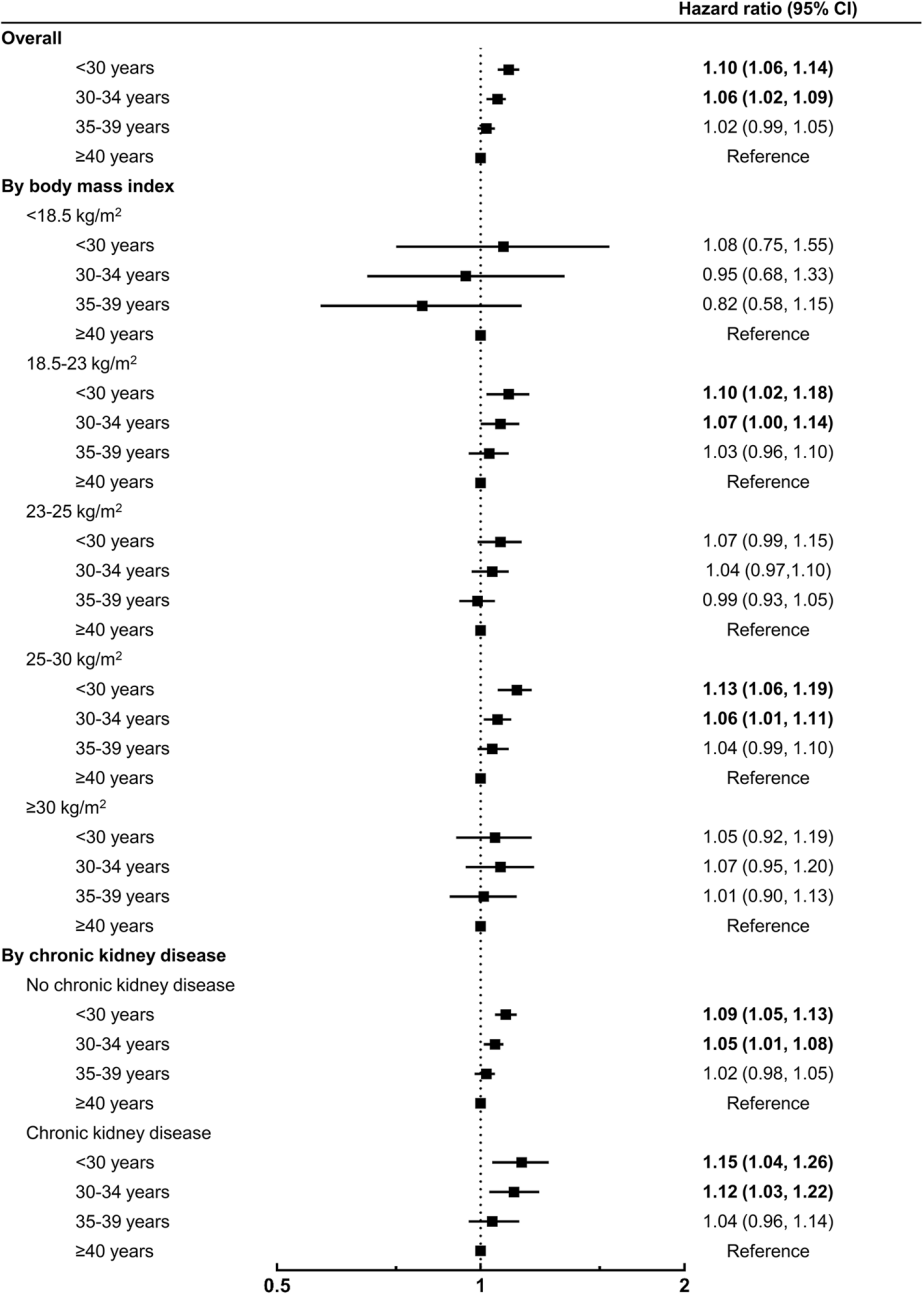
Adjusted hazard ratios and 95% confidence intervals (CIs) for incident gout according to reproductive span. Multivariable model included age, reproductive span, oral contraceptives, hormone replacement therapy, parity, breastfeeding, body mass index, smoking, alcohol consumption, regular exercise, hypertension, diabetes mellitus, hyperlipidemia, chronic kidney disease, and income

In multivariable models, late age at menarche, early age at menopause, and short reproductive span were associated with an increased risk of gout (Table 2). Women who experienced menarche after age of 16 had a higher risk of gout (adjusted HR [aHR] 1.10, 95% CI 1.02–1.19) than women who experienced menarche before age of 12. Compared to women who were 50–54 years of age at menopause, the risk of gout was higher in women who had menopause before the age of 50 (< 40 years [aHR 1.12, 95% CI 1.06–1.19], 40–44 yeras [aHR 1.06, 95% CI 1.02–1.10] and 45–49 years [aHR 1.03, 95% CI 1.01–1.04]) and lower in women who had menopause after the age of 55 (aHR 0.97, 95% CI 0.94–0.99). When compared to women with a reproductive span ≥40 years, those with a reproductive span < 35 years had a higher risk of gout: < 30 years (aHR 1.10, 95% CI 1.06–1.14) and 30–34 years (aHR 1.06, 95% CI 1.02–1.09) (Fig. 2).

**Table 2:** Hazard ratios and 95% confidence intervals for incident gout according to various reproductive factors

	Subjects (n)	Events (n)	Follow-up duration (PYs)	Incidence rate (per 1000 PYs)	Hazard ratio (95% confidence interval)		
					Model 1	Model 2	Model 3
Age at menarche (years)							
≤ 12	11,847	635	96,616	6.57	1 (Ref.)	1 (Ref.)	
13–14	146,020	8329	1,188,213	7.01	1.07 (0.98–1.16)	1.06 (0.98–1.15)	
15–16	430,498	24,984	3,505,757	7.13	**1.08 (1.00**–**1.17)**	1.07 (0.99–1.16)	
> 16	488,013	30,104	3,975,279	7.57	**1.15 (1.06**–**1.24)**	**1.10 (1.02**–**1.19)**	
Age at menopause (years)							
< 40	15,754	1091	127,654	8.55	**1.19 (1.12**–**1.26)**	**1.12 (1.06**–**1.19)**	
40–44	53,742	3412	436,253	7.82	**1.09 (1.05**–**1.13)**	**1.06 (1.02**–**1.10)**	
45–49	290,992	17,389	2,371,231	7.33	**1.02 (1.00**–**1.04)**	**1.03 (1.01**–**1.04)**	
50–54	597,465	34,968	4,867,834	7.18	1 (Ref.)	1 (Ref.)	
≥ 55	118,425	7192	962,894	7.47	**1.04 (1.01**–**1.07)**	**0.97 (0.94**–**0.99)**	
Reproductive span (years)							
< 30	129,369	8259	1,051,444	7.85	**1.06 (1.02**–**1.10)**		**1.10 (1.06–1.14)**
30–34	435,768	26,066	3,551,041	7.34	0.98 (0.96–1.02)		**1.06 (1.02–1.09)**
35–39	439,093	25,375	3,577,359	7.09	**0.95 (0.92**–**0.99)**		1.02 (0.99–1.05)
≥ 40	72,148	4352	586,022	7.43	1 (Ref.)		1 (Ref.)
Oral contraceptives (years)							
Never	895,102	52,498	7,292,924	7.20	1 (Ref.)	1 (Ref.)	1 (Ref.)
< 1	110,098	6846	895,403	7.65	**1.06 (1.04**–**1.09)**	**1.03 (1.00**–**1.06)**	**1.03 (1.01–1.06)**
≥ 1	71,178	4708	577,539	8.15	**1.13 (1.10**–**1.17)**	**1.05 (1.02**–**1.08)**	**1.05 (1.02–1.08)**
Hormone replacement therapy (years)							
Never	877,088	51,071	7,142,979	7.15	1 (Ref.)	1 (Ref.)	1 (Ref.)
< 2	116,184	7508	946,434	7.93	**1.11 (1.08**–**1.14)**	**1.16 (1.13**–**1.18)**	**1.16 (1.13–1.18)**
2–5	47,584	3075	387,867	7.93	**1.11 (1.07**–**1.15)**	**1.16 (1.12**–**1.21)**	**1.16 (1.12–1.21)**
≥ 5	35,522	2398	288,586	8.31	**1.16 (1.12**–**1.21)**	**1.19 (1.14**–**1.23)**	**1.18 (1.14–1.23)**
Parity							
0 children	28,422	1700	230,168	7.39	1 (Ref.)	1 (Ref.)	1 (Ref.)
1 child	73,227	4210	595,161	7.07	0.96 (0.91–1.01)	1.00 (0.94–1.06)	1.00 (0.94–1.06)
≥ 2 children	974,729	58,142	7,940,536	7.32	0.99 (0.94–1.04)	0.99 (0.94–1.05)	0.99 (0.94–1.05)
Breastfeeding (months)							
Never	79,504	4619	644,896	7.16	1 (Ref.)	1 (Ref.)	1 (Ref.)
< 6	78,178	4070	637,261	6.39	**0.89 (0.85**–**0.93)**	**0.93 (0.89**–**0.98)**	**0.93 (0.89–0.97)**
6–12	199,070	11,213	1,621,738	6.91	**0.96 (0.93**–**1.00)**	0.98 (0.94–1.01)	0.98 (0.94–1.01)
≥ 12	719,626	44,150	5,861,970	7.53	**1.05 (1.02**–**1.08)**	1.00 (0.96–1.03)	1.00 (0.96–1.03)

*PYs* person-years, *Ref.* reference group

Model 1: crude model

Model 2: multivariable model including age, age at menarche, age at menopause, oral contraceptives, hormone replacement therapy, parity, breastfeeding, body mass index, smoking, alcohol consumption, regular exercise, hypertension, diabetes mellitus, hyperlipidemia, chronic kidney disease, and income

Model 3: multivariable model including variables of Model 2 except for age at menarche and age at menopause replaced by reproductive span

### Association between incident gout and exogenous estrogen related factors

The risk of incident gout was higher in those who used OC compared to women who never used OC (aHR 1.03, 95% CI 1.00–1.06 in those who used OC < 1 year vs. aHR 1.05, 95% CI 1.02–1.08 in those who used OC ≥1 year). The use of HRT was also associated with an increased risk of gout, and the risk was highest among those who used HRT for > 5 years (aHR 1.19, 95% CI 1.14–1.23).

### Association between incident gout and pregnancy related factors

Parity did not affect the risk of gout. Compared with subjects who had never breastfed, those who had breastfed for < 6 months had a lower risk of gout (aHR 0.93, 95% CI 0.89–0.97). However, those who breastfed for ≥6 months had no association with a risk of gout (aHR 0.98, 95% CI 0.94–1.01 in those who breastfed for 6–12 months vs. aHR 1.00, 95% CI 0.96–1.03 for those who breastfed for ≥12 months).

### Stratified analysis

Table 3 shows the effect of reproductive factors on the risk of incident gout in women stratified by their BMI. The association between age at menarche and incident gout differed according to the BMI group (*p* for interaction = 0.0033). Late age at menarche was most clearly associated with the risk of gout in the overweight group (aHR 1.30, 95% CI 1.09–1.55 in those aged 13–14 years vs. aHR 1.30, 95% CI 1.09–1.54 in those aged 15–16 years vs. aHR 1.34, 95% CI 1.13–1.60 in those > 16 years). The risk was not statistically significant in the normal and underweight groups, but the trend of increasing risk with age at menarche was still observed. However, in the obese and severely obese groups, there was no association between age at menarche and the risk of gout. The association between other reproductive factors and incident gout was not affected by BMI (*p* for interaction > 0.05).

**Table 3:** Hazard ratios and 95% confidence intervals for association between incident gout and various reproductive factors in women stratified by body mass index

	Body mass index (kg/m^**2**^)					***p*** for interaction
	< 18.5	18.5–23	23–25	25–30	≥30	
Age at menarche (years)						**0.0033**
≤ 12	1 (Ref.)	1 (Ref.)	1 (Ref.)	1 (Ref.)	1 (Ref.)	
13–14	1.04 (0.48–2.25)	1.01 (0.87–1.16)	**1.30 (1.09**–**1.55)**	1.01 (0.88–1.14)	0.92 (0.69–1.22)	
15–16	1.39 (0.66–2.95)	1.08 (0.93–1.24)	**1.30 (1.09**–**1.54)**	0.96 (0.85–1.09)	0.91 (0.69–1.20)	
> 16	1.38 (0.65–2.93)	1.11 (0.96–1.28)	**1.34 (1.13**–**1.60)**	1.00 (0.88–1.13)	0.94 (0.71–1.24)	
Age at menopause (years)						0.6741
< 40	1.31 (0.82–2.07)	**1.20 (1.07**–**1.34)**	1.05 (0.92–1.20)	1.10 (0.99–1.21)	1.17 (0.95–1.43)	
40–44	1.29 (1.00–1.68)	1.04 (0.97–1.11)	1.06 (0.99–1.14)	**1.09 (1.03**–**1.16)**	0.94 (0.83–1.08)	
45–49	0.98 (0.83–1.15)	1.03 (1.00–1.07)	1.03 (0.99–1.06)	1.02 (0.99–1.06)	1.01 (0.94–1.09)	
50–54	1 (Ref.)	1 (Ref.)	1 (Ref.)	1 (Ref.)	1 (Ref.)	
≥ 55	0.97 (0.73–1.29)	0.98 (0.93–1.03)	0.98 (0.93–1.03)	**0.95 (0.92**–**0.99)**	0.95 (0.86–1.04)	
Reproductive span						0.5289
< 30	1.08 (0.75, 1.55)	**1.10 (1.02, 1.18)**	1.07 (0.99, 1.15)	**1.13 (1.06, 1.19)**	1.05 (0.92, 1.19)	
30–34	0.95 (0.68, 1.33)	**1.07 (1.00, 1.14)**	1.04 (0.97,1.10)	**1.06 (1.01, 1.11)**	1.07 (0.95, 1.20)	
35–39	0.82 (0.58, 1.15)	1.03 (0.96, 1.10)	0.99 (0.93, 1.05)	1.04 (0.99, 1.10)	1.01 (0.90, 1.13)	
≥ 40	1 (Ref.)	1 (Ref.)	1 (Ref.)	1 (Ref.)	1 (Ref.)	
Oral contraceptives (years)						0.1990
Never	1 (Ref.)	1 (Ref.)	1 (Ref.)	1 (Ref.)	1 (Ref.)	
< 1	1.12 (0.88–1.43)	1.04 (0.99–1.09)	1.01 (0.96–1.06)	**1.04 (1.00–1.08)**	1.00 (0.91–1.11)	
≥ 1	1.13 (0.83–1.54)	**1.12 (1.06**–**1.18)**	1.04 (0.98–1.10)	1.01 (0.96–1.06)	1.05 (0.94–1.18)	
Hormone replacement therapy (years)						0.4493
Never	1 (Ref.)	1 (Ref.)	1 (Ref.)	1 (Ref.)	1 (Ref.)	
< 2	1.22 (0.99–1.52)	**1.19 (1.14**–**1.24)**	**1.16 (1.10**–**1.21)**	**1.13 (1.09**–**1.18)**	1.11 (0.99–1.24)	
2–5	0.91 (0.63–1.31)	**1.17 (1.10**–**1.24)**	**1.19 (1.11**–**1.28)**	**1.15 (1.08**–**1.23)**	1.11 (0.91–1.35)	
≥ 5	1.01 (0.67–1.53)	**1.21 (1.13**–**1.30)**	**1.21 (1.12**–**1.31)**	**1.15 (1.07**–**1.24)**	1.16 (0.89–1.40)	
Parity						0.9025
0 children	1 (Ref.)	1 (Ref.)	1 (Ref.)	1 (Ref.)	1 (Ref.)	
1 child	1.36 (0.88–1.96)	0.98 (0.89–1.09)	1.03 (0.91–1.15)	0.98 (0.88–1.08)	0.94 (0.73–1.21)	
≥ 2 children	1.11 (0.76–1.64)	0.96 (0.87–1.05)	1.02 (0.92–1.13)	0.99 (0.91–1.09)	1.00 (0.80–1.26)	
Breastfeeding (months)						0.6125
Never	1 (Ref.)	1 (Ref.)	1 (Ref.)	1 (Ref.)	1 (Ref.)	
< 6	0.84 (0.62–1.14)	1.00 (0.93–1.08)	**0.90 (0.82**–**0.98)**	**0.90 (0.83**–**0.98)**	0.91 (0.74–1.11)	
6–12	0.81 (0.61–1.08)	1.02 (0.95–1.08)	0.96 (0.89–1.03)	0.95 (0.89–1.02)	0.95 (0.81–1.12)	
≥ 12	0.91 (0.71–1.18)	1.05 (0.99–1.12)	0.97 (0.91–1.04)	0.97 (0.91–1.03)	0.95 (0.82–1.10)	

Multivariable model including age, age at menarche, age at menopause, oral contraceptives, hormone replacement therapy, parity, breastfeeding, body mass index, smoking, alcohol consumption, regular exercise, hypertension, diabetes mellitus, hyperlipidemia, chronic kidney disease, and income. For the analysis of the reproductive span, a modified version of multivariable model – in which age at menarche and age at menopause were replaced by reproductive span – was used

*Ref.* reference group

In a stratified analysis according to the presence/absence CKD, the association between age at menarche, age at menopause, reproductive span, parity, and gout risk did not differ between the groups (Table 4). Breastfeeding for > 6 months in the CKD group was associated with a low risk of incident gout (aHR 0.86, 95% CI 0.77–0.97 in those who had breastfed for 6–12 months vs. aHR 0.89, 95% CI 0.80–0.98 in those who had breastfed for ≥12 months). OC use for > 1 year (aHR 1.05, 95% CI 1.01–1.08) and HRT (aHR 1.17, 95% CI 1.14–1.20 with usage for < 2 years vs. aHR 1.18, 95% CI 1.13–1.22 with usage for 2–5 years vs. aHR 1.23, 95% CI 1.17–1.28 with usage for ≥5 years) showed a high risk of incident gout only in the group without CKD.

**Table 4:** Hazard ratios and 95% confidence intervals for association between incident gout and various reproductive factors in women stratified by the presence/absence of chronic kidney disease

	No CKD	CKD	***p*** for interaction
Age at menarche (years)			0.4141
≤ 12	1 (Ref.)	1 (Ref.)	
13–14	1.07 (0.98–1.16)	1.02 (0.80–1.29)	
15–16	1.06 (0.98–1.16)	1.09 (0.86–1.38)	
> 16	1.10 (1.01–1.20)	1.11 (0.87–1.40)	
Age at menopause (years)			0.4028
< 40	**1.10 (1.03**–**1.18)**	**1.23 (1.06**–**1.42)**	
40–44	**1.06 (1.02**–**1.10)**	1.05 (0.96–1.15)	
45–49	**1.02 (1.00**–**1.04)**	**1.06 (1.01**–**1.11)**	
50–54	1 (Ref.)	1 (Ref.)	
≥ 55	**0.97 (0.94**–**1.00)**	0.95 (0.89–1.02)	
Reproductive span			0.3525
< 30	**1.09 (1.05, 1.13)**	**1.15 (1.04, 1.26)**	
30–34	**1.05 (1.01, 1.08)**	**1.12 (1.03, 1.22)**	
35–39	1.02 (0.98, 1.05)	1.04 (0.96, 1.14)	
≥40	1 (Ref.)	1 (Ref.)	
Oral contraceptives (years)			0.7684
Never	1 (Ref.)	1(Ref.)	
< 1	1.03 (1.00–1.05)	1.07 (0.99–1.14)	
≥ 1	**1.05 (1.01**–**1.08)**	1.06 (0.98–1.15)	
Hormone replacement therapy (years)			**<.0001**
Never	1 (Ref.)	1 (Ref.)	
< 2	**1.17 (1.14**–**1.20)**	1.06 (0.99–1.15)	
2–5	**1.18 (1.13**–**1.22)**	1.08 (0.97–1.20)	
≥ 5	**1.23 (1.17**–**1.28)**	0.97 (0.86–1.08)	
Parity			**0.0296**
0 children	1 (Ref.)	1 (Ref.)	
1 child	1.02 (0.96–1.08)	0.86 (0.72–1.02)	
≥ 2 children	0.99 (0.94–1.05)	1.00 (0.85–1.16)	
Breastfeeding (months)			0.1159
Never	1 (Ref.)	1 (Ref.)	
< 6	**0.94 (0.89**–**0.98)**	0.90 (0.79–1.02)	
6–12	0.99 (0.95–1.03)	**0.86 (0.77**–**0.97)**	
≥ 12	1.01 (0.97–1.05)	**0.89 (0.80**–**0.98)**	

Multivariable model including age, age at menarche, age at menopause, oral contraceptives, hormone replacement therapy, parity, breastfeeding, body mass index, smoking, alcohol consumption, regular exercise, hypertension, diabetes mellitus, hyperlipidemia, chronic kidney disease, and income. For the analysis of the reproductive span, a modified version of multivariable model – in which age at menarche and age at menopause were replaced by reproductive span – was used

*Ref.* reference group

## Discussion

In this large nationwide population-based cohort of postmenopausal women, late menarche, early menopause, and short reproductive span were associated with a high risk of incident gout. The use of OC and HRT was also associated with an increased risk of gout. The association between reproductive factors and gout was relatively low in severely obese women and in women with CKD.

Our results are consistent with those of Nurses’ Health Study, which had shown that early menopause was associated with an increased risk of incident gout [7]. Estrogen may play a protective role in hyperuricemia and gout by promoting renal clearance of uric acid [3, 15]. A cross-sectional study in Germany had also demonstrated that earlier age at menarche and postmenopausal status were related to a higher serum uric acid concentration [5]. In a cross-sectional study of 58,870 middle-aged Korean women, the prevalence of hyperuricemia had increased with menopausal transition [16]. This suggests that the premenopausal level of estrogen acts on the serum uric acid level and lowers the risk of gout. In addition, since estrogen promotes the progression of macrophages to the IL10-dependent acquired deactivation phase and may have an anti-inflammatory effect, changes in estrogen levels may affect the onset of inflammatory diseases such as gout [17].

OC use was associated with a high risk of gout. In the KORA F4 study, current use of OC was not related to uric acid level, but past use of OC was related to a high uric acid concentration [5]. Since our study included only postmenopausal women, all the subjects who used OC corresponded to the past users of OC. This is consistent with the results of previous studies. Further research is needed to evaluate how exposure to exogenous estrogen at a young age is associated with increased gout risk and uric acid levels in postmenopausal women.

In this study, HRT increased the risk of incident gout. This contradicts the results of the Nurses’ Health Study, which had suggested that current HRT use is associated with a reduced risk of incident gout [7], and the Heart and Estrogen-Progestin Replacement Study [18] and a recent retrospective cohort study [19], those that had suggested that estrogen plus progestin therapy was associated with a decrease in uric acid levels. Previous studies had classified HRT use into never, past, and current use, so it is impossible to directly compare it with our study, which classified HRT by period of use. However, this difference might have occurred because adverse events, such as lipid changes related to HRT use, might have affected the occurrence of gout. As another possibility, since HRT users suffer from estrogen deficiency, their increased gout risk might have actually been due to the effect of estrogen deficiency and not that of HRT. Because there was a difference in the role of HRT on gout between previous studies and our study, caution is needed in interpreting the results of our study, and additional research results are needed to confirm the effect of HRT on gout.

In our study, parity was not related to incident gout. This may be interpreted in line with the KORA F4 study, which had shown no association between hyperuricemia and parity in 1530 women aged 32–81 years in Southern Germany [5]. Previous studies have suggested that parity is associated with the risk of various diseases like cardiovascular diseases, and that hormonal and metabolic changes related to pregnancy are the possible mechanisms for this [20, 21]. In the case of gout, studies – including ours – have shown that there is no association between gout and parity, but further studies are needed to confirm this. During pregnancy, maternal plasma estradiol levels rise progressively [22]. Estrogen is mainly produced in the corpus luteum before pregnancy and in the very early stages of pregnancy, but after 9 weeks of pregnancy when the hormonal ovary-to-placenta shift occurs, placenta becomes the main production site of estrogen [23]. In the early postpartum period after pregnancy, the ovarian function is suppressed, and in most non-lactating women, ovulation does not occur until 6 weeks after delivery [24]. One cross-sectional study showed that women who delivered within 3 years had lower urinary estradiol metabolite concentrations compared to nulliparous women [25]. There was a study showing that parity is inversely associated with free estradiol levels in postmenopausal women [26]. These findings suggest that parity is associated with increased exposure to high-level endogenous estrogen during pregnancy, but may be associated with decreased exposure to endogenous estrogen thereafter. Possible explanations for the lack of association between parity and incident gout in our study are that the change in estrogen level due to parity was not sufficient to affect the occurrence of gout, or that differences in other factors related parity, such as health-related behaviors related to parity and child rearing, or medical conditions of parous women, acted as confounding factors in the occurrence of gout.

To our knowledge, our study was the first to explore the association between breastfeeding and gout, and our results suggest that breastfeeding for < 6 months is associated with a low risk of incident gout. There was no significant association with the risk of gout when the breastfeeding period was > 6 months. The possible mechanisms for the different effects on gout depending on the duration of breastfeeding are as follows. Since breastfeeding promotes postpartum weight loss, short-term breastfeeding may play a protective role in the development of gout through weight loss [27]. Long-term breastfeeding inhibits ovulation and causes gonadotropin inhibition, resulting in decreased plasma estradiol production [28]. Estrogen also plays a protective role in hyperuricemia, so prolonged breastfeeding may not have a protective effect on gout. However, the degree of association between breastfeeding and gout was not strong in our study, and when other reproductive factors had been adjusted for, the association tended to weaken. Accordingly, it is possible that other factors that had not been adjusted for might have played a confounding role.

A stratified analysis showed that the association between reproductive factors and gout was relatively weak in the severely obese and CKD groups. Obesity and CKD are well-known risk factors for gout [29–31], and it is possible that reproductive factors have a relatively small effect on gout incidence in women with these risk factors. In addition, since the adipose tissue metabolizes and secretes sex hormones [32], it is possible that estrogen deficiency in postmenopausal women is relatively alleviated in obese patients and that the effect of reproductive factors is small.

Our study had several limitations. First, information on reproductive factors was collected after the age of 40 years, so there was a potential for recall bias. Second, because the information had been collected in advance through the questionnaire provided by the national health screening program, the classification of items was limited, and information such as the type or dose of HRT and OC could not be obtained. In the case of OC, since only combined OCs are used in Korea except for the morning-after pill, it can be estimated that most OC users are combined OC users. However, for HRT, various doses, formulations, and ingredients such as combined pills of estrogen and progestin or estrogen only are used, and there have been previous studies that showed different effects on uric acid depending on the ingredients [6, 19]. So the inability to include detailed HRT information in the analysis was an important limitation of our study. Third, since uric acid level was not included in the health examination items, the effect of reproductive factors on uric acid level could not be investigated. Fourth, the inclusion of various reproductive factors in a model would introduce risk of collinearity and confounding. However, when the variation inflation factor (VIF), which is a measure of the amount of multicollinearity, was calculated, all reproductive variables had VIF values of 1.0 or 1.1, corresponding to VIF < 2.5, which is a conservative level without concern of multicollinearity. Also, when separate analyzes according to variables were performed according to the time order of reproductive factors, the association between reproductive factors and incident gout was observed in the same way (Supplementary Table S1-S5). Despite these limitations, our study is meaningful in that we studied the association between the occurrence of gout and various reproductive factors in a large nationwide population-based cohort of postmenopausal women.

Our study showed conflicting results in that exposure to endogenous female sex hormones reduced the risk of gout, whereas exposure to exogenous hormones increased the risk of gout. Although the cause of this cannot be clearly explained, it is expected that endogenous and exogenous hormones have different physiological effects on the body. The results of studies that have suggested that the natural menstrual cycle and OC influence renal electrolyte handling differently [33], and that exogenous progesterone inhibits the hypothalamic-pituitary-adrenal axis (unlike endogenous progesterone) [34] increase the reliability of this explanation. Further research is needed on how endogenous and exogenous hormones may act differently with respect to uric acid metabolism and the development of gout.

## Conclusions

We found that shorter exposure to endogenous estrogen was associated with an increased risk of incident gout. Exposure to exogenous estrogens, such as OC and HRT, was associated with a high risk of incident gout. However, this association was weak in severely obese women and in women with CKD. Subsequent research is needed to further identify the mechanisms by which reproductive factors influence the development of gout.

## Supplementary Information

**Additional file 1: Supplementary Table S1.** Hazard ratios and 95% confidence intervals for incident gout according to age at menarche. **Supplementary Table S2.** Hazard ratios and 95% confidence intervals for incident gout according to parity and oral contraceptives use. **Supplementary Table S3.** Hazard ratios and 95% confidence intervals for incident gout according to parity, breastfeeding and oral contraceptives use who had parity (*n* = 1,047,956). **Supplementary Table S4.** Hazard ratios and 95% confidence intervals for incident gout according to age at menopause. **Supplementary Table S5.** Hazard ratios and 95% confidence intervals for incident gout according to hormone replacement therapy.

## Data Availability

The data that support the findings of this study are available from the NHIS but restrictions apply to the availability of these data, which were used under license for the current study, and so are not publicly available. Data are however available from the authors upon reasonable request and with permission of NHIS.

## Abbreviations

OC: Oral contraceptive
HRT: Hormone replacement therapy
NHANES: National Health and Nutrition Examination Survey
NHIS: National Health Insurance Service
NCSP: National Cancer Screening Program
ICD-10: International Classification of Disease 10th revision
BMI: Body mass index
eGFR: Estimated glomerular filtration rate
CKD: Chronic kidney disease
SD: Standard deviation
HRs: Hazard ratios
95% CIs: 95% confidence intervals
aHR: Adjusted hazard ratio
