# Supplementary material for: Association between female reproductive factors and gout: a nationwide population-based cohort study of 1 million postmenopausal women
Source: Arthritis Res Ther. 2021 Dec 16;23:304. doi: 10.1186/s13075-021-02701-w (PMC8675498; doi:10.1186/s13075-021-02701-w)
Supplement: Supplementary file 1 — Additional file 1: Supplementary Table S1. Hazard ratios and 95% confidence intervals for incident gout according to age at menarche. Supplementary Table S2. Hazard ratios and 95% confidence intervals for incident gout according to parity and oral contraceptives use. Supplementary Table S3. Hazard ratios and 95% confidence intervals for incident gout according to parity, breastfeeding and oral contraceptives use who had parity (n = 1,047,956). Supplementary Table S4. Hazard ratios and 95% confidence intervals for incident gout according to age at menopause. Supplementary Table S5. Hazard ratios and 95% confidence intervals for incident gout according to hormone replacement therapy. [file 13075_2021_2701_MOESM1_ESM.pdf]

Supplementary Table S1 Hazard ratios and 95% confidence intervals for incident gout according to age at menarche

Reproductive factors	Subjects (N)	Events (n)	Follow-up duration (PYs)	IR	HR (95% CI)*
Age at menarche (years)					
≤12	11,847	635	96,616	6.57	1 (Ref.)
13-14	146,020	8,329	1,188,213	7.01	1.06 (0.97−1.15)
15-16	430,498	24,984	3,505,757	7.13	1.06 (0.98−1.14)
>16	488,013	30,104	3,975,279	7.57	**1.09 (1.01−1.18)**
p for trend					**<.0001**

PY, person-years; IR, incidence rate per 10,000 person-years; HR, hazard ratio; CI, confidence interval; Ref., reference group

*Multivariable model included age, age at menarche, body mass index, smoking, alcohol consumption, regular exercise, hypertension, diabetes mellitus, hyperlipidemia, chronic kidney disease, and income.

Supplementary Table S2 Hazard ratios and 95% confidence intervals for incident gout according to parity and oral contraceptives use

Reproductive factors	Subjects (N)	Events (n)	Follow-up duration (PYs)	IR	HR (95% CI)*
Oral contraceptives (years)					
Never	895,102	52,498	7,292,924	7.20	1 (Ref.)
<1	110,098	6,846	895,403	7.65	**1.05 (1.02−1.07)**
≥1	71,178	4,708	577,539	8.15	**1.07 (1.04−1.10)**
p for trend					**<.0001**
Parity					
0 children	28,422	1,700	230,168	7.39	1 (Ref.)
1 child	73,227	4,210	595,161	7.07	0.98 (0.93−1.04)
≥2 children	974,729	58,142	7,940,536	7.32	0.97 (0.92−1.02)
p for trend					0.9295

PY, person-years; IR, incidence rate per 10,000 person-years; HR, hazard ratio; CI, confidence interval; Ref., reference group

*Multivariable model included age, age at menarche, oral contraceptives, parity, body mass index, smoking, alcohol consumption, regular exercise, hypertension, diabetes mellitus, hyperlipidemia, chronic kidney disease, and income.

Supplementary Table S3 Hazard ratios and 95% confidence intervals for incident gout according to parity, breastfeeding and oral contraceptives use who had parity (n = 1,047,956)

Reproductive factors	Subjects (N)	Events (n)	Follow-up duration (PYs)	IR	HR (95% CI)*
Oral contraceptives (years)					
Never	870351	51041	7092479.39	7.20	1 (Ref.)
<1	108131	6716	879496.97	7.64	**1.05 (1.02−1.07)**
≥1	69474	4595	563721.26	8.15	**1.07 (1.04−1.10)**
p for trend					**<.0001**
Parity					
1 child	73227	4210	595161.47	7.07	1 (Ref.)
≥2 children	974729	58142	7940536.15	7.32	0.99 (0.95−1.02)
p for trend					0.3960
Breastfeeding (months)					
Never	58981	3424	479148.99	7.15	1 (Ref.)
<6	77216	4014	629367.16	6.38	**0.93 (0.89−0.97)**
6-12	197091	11082	1605635.33	6.90	0.96 (0.93−1.00)
≥12	714668	43832	5821546.13	7.53	0.98 (0.95−1.02)
p for trend					**<.0001**

PY, person-years; IR, incidence rate per 10,000 person-years; HR, hazard ratio; CI, confidence interval; Ref., reference group

*Multivariable models included age, age at menarche, oral contraceptives, parity, breastfeeding, body mass index, smoking, alcohol consumption, regular exercise, hypertension, diabetes mellitus, hyperlipidemia, chronic kidney disease, and income.

Supplementary Table S4 Hazard ratios and 95% confidence intervals for incident gout according to age at menopause

Reproductive factors	Subjects (N)	Events (n)	Follow-up duration (PYs)	IR	HR (95% CI)*
Age at menopause (years)					
<40	15,754	1,091	127,654	8.55	**1.16 (1.08−1.22)**
40-44	53,742	3,412	436,253	7.82	**1.08 (1.04−1.12)**
45-49	290,992	17,389	2,371,231	7.33	**1.04 (1.02−1.06)**
50-54	597,465	34,968	4,867,834	7.18	1 (Ref.)
≥55	118,425	7,192	962,894	7.47	**0.97 (0.94−0.99)**
p for trend					**<.0001**

PY, person-years; IR, incidence rate per 10,000 person-years; HR, hazard ratio; CI, confidence interval; Ref., reference group

*Multivariable models includes age, age at menarche, age at menopause, oral contraceptives, parity, breastfeeding, body mass index, smoking, alcohol consumption, regular exercise, hypertension, diabetes mellitus, hyperlipidemia, chronic kidney disease, and income.

Supplementary Table S5 Hazard ratios and 95% confidence intervals for incident gout according to hormone replacement therapy

Reproductive factors	Subjects (N)	Events (n)	Follow-up duration (PYs)	IR	HR (95% CI)*
Hormone replacement therapy (years)					
Never	877,088	51,071	7,142,979	7.15	1 (Ref.)
<2	116,184	7,508	946,434	7.93	**1.16 (1.13−1.18)**
2-5	47,584	3,075	387,867	7.93	**1.16 (1.12−1.21)**
≥5	35,522	2,398	288,586	8.31	**1.19 (1.14−1.23)**
p for trend					**<.0001**

PY, person-years; IR, incidence rate per 10,000 person-years; HR, hazard ratio; CI, confidence interval; Ref., reference group

*Multivariable model includes age, age at menarche, age at menopause, oral contraceptives, hormone replacement therapy, parity, breastfeeding, body mass index, smoking, alcohol consumption, regular exercise, hypertension, diabetes mellitus, hyperlipidemia, chronic kidney disease, and income.
